# Assessing vertebrate biodiversity in a kelp forest ecosystem using environmental DNA

**DOI:** 10.1111/mec.13481

**Published:** 2015-12-24

**Authors:** Jesse A. Port, James L. O'Donnell, Ofelia C. Romero‐Maraccini, Paul R. Leary, Steven Y. Litvin, Kerry J. Nickols, Kevan M. Yamahara, Ryan P. Kelly

**Affiliations:** ^1^ Center for Ocean SolutionsStanford UniversityStanfordCA94305USA; ^2^ School of Marine and Environmental AffairsUniversity of WashingtonSeattleWA98105USA; ^3^ Hopkins Marine StationStanford UniversityPacific GroveCA93950USA; ^4^ Monterey Bay Aquarium Research InstituteMoss LandingCA95039USA; ^5^ California State UniversityMonterey BaySeasideCA93955USA

**Keywords:** biodiversity, eDNA, fish, metabarcoding, monitoring, next generation sequencing

## Abstract

Preserving biodiversity is a global challenge requiring data on species’ distribution and abundance over large geographic and temporal scales. However, traditional methods to survey mobile species’ distribution and abundance in marine environments are often inefficient, environmentally destructive, or resource‐intensive. Metabarcoding of environmental DNA (eDNA) offers a new means to assess biodiversity and on much larger scales, but adoption of this approach for surveying whole animal communities in large, dynamic aquatic systems has been slowed by significant unknowns surrounding error rates of detection and relevant spatial resolution of eDNA surveys. Here, we report the results of a 2.5 km eDNA transect surveying the vertebrate fauna present along a gradation of diverse marine habitats associated with a kelp forest ecosystem. Using PCR primers that target the mitochondrial 12S rRNA gene of marine fishes and mammals, we generated eDNA sequence data and compared it to simultaneous visual dive surveys. We find spatial concordance between individual species’ eDNA and visual survey trends, and that eDNA is able to distinguish vertebrate community assemblages from habitats separated by as little as ~60 m. eDNA reliably detected vertebrates with low false‐negative error rates (1/12 taxa) when compared to the surveys, and revealed cryptic species known to occupy the habitats but overlooked by visual methods. This study also presents an explicit accounting of false negatives and positives in metabarcoding data, which illustrate the influence of gene marker selection, replication, contamination, biases impacting eDNA count data and ecology of target species on eDNA detection rates in an open ecosystem.

## Introduction

Environmental conservation, management and basic ecology require information on species distributions and trends in abundance. However, for many species and ecosystems—especially in freshwater and marine environments and for mobile organisms—practical methods to monitor biodiversity are often inefficient (e.g. visual surveys), environmentally destructive (e.g. trawls) or require significant person‐time and resources (Jones 1992; Baldwin *et al*. 1996; Wheeler *et al*. 2004). Furthermore, given the continuous decline in global biodiversity (Butchart *et al*. 2010), a sampling technique that targets communities instead of individual species—and over larger geographic and temporal scales—could enable more comprehensive management and greatly improved resolution for core ecological research.

Environmental DNA (eDNA) offers a high‐throughput, potentially cheaper, vastly more sensitive, and less invasive approach to survey biodiversity than conventional methods. eDNA is genetic material obtained directly from environmental samples (water, sediment, soil, etc.) that is derived from microbes or shed from multicellular organisms (Taberlet *et al*. 2012). While eDNA has commonly been used to assess microbial diversity and abundance (Venter *et al*. 2004; Rusch *et al*. 2007), only recently has the technique been used to survey higher eukaryotes including fishes (Thomsen *et al*. 2012a,b; Jerde *et al*. 2013), mammals (Andersen *et al*. 2012; Foote *et al*. 2012), amphibians (Ficetola *et al*. 2008; Pilliod *et al*. 2014) and invertebrates (Goldberg *et al*. 2013; Deiner & Altermatt 2014; Mächler *et al*. 2014). The majority of these macrobial eDNA investigations to date have been species‐specific, but multi‐species PCR in combination with high‐throughput sequencing (i.e. metabarcoding) can reveal whole‐community eDNA. eDNA metabarcoding has been used for such applications as characterizing animal diet content (Deagle *et al*. 2010; Shehzad *et al*. 2012), screening for the presence of invasive species in the bait trade (Mahon *et al*. 2014), and to a lesser extent biodiversity profiling (Thomsen *et al*. 2012a,b), but uses of this technique for field ecology and conservation have lagged due to unknown error rates of detection and spatial resolution of eDNA in the field.

Small volumes of water contain eDNA sufficient to reliably detect target organisms—including invasive, endangered and rare species—in freshwater environments (Jerde *et al*. 2011, 2013; Thomsen *et al*. 2012b; Goldberg *et al*. 2013; Takahara *et al*. 2013; Mächler *et al*. 2014; Laramie *et al*. 2015; Spear *et al*. 2015), but few such marine studies exist (Foote *et al*. 2012; Thomsen *et al*. 2012a; Kelly *et al*. 2014; Miya *et al*. 2015). The ocean imposes an additional set of physical and chemical constraints affecting eDNA distribution and detection probability; currents, tides, wind and salinity all impact eDNA degradation rates and persistence in seawater (Thomsen *et al*. 2012a; Barnes *et al*. 2014). Nevertheless, where organisms’ generation of eDNA outpaces the combined forces of degradation and transport we expect to see a significant biological signal of species living in sampled habitats.

Here, we present data from a field survey of marine vertebrates in a coastal ecosystem in Monterey Bay, CA using both eDNA and conventional visual methods. The study area spanned a diversity of distinct habitat types, including kelp forests, which are highly productive epicentres of biodiversity and are quintessential sites of marine ecological research and conservation (Dayton 1985; Steneck *et al*. 2002). Our objectives were: (i) to compare the performance of eDNA relative to conventional visual surveys, (ii) to test for trends in abundance of taxon‐specific eDNA across multiple habitats, (iii) to compare eDNA community composition among habitats and (iv) to estimate the relevant spatial scale of community‐level eDNA sampling.

## Materials and methods

### Visual fish surveys

**Figure 1:**
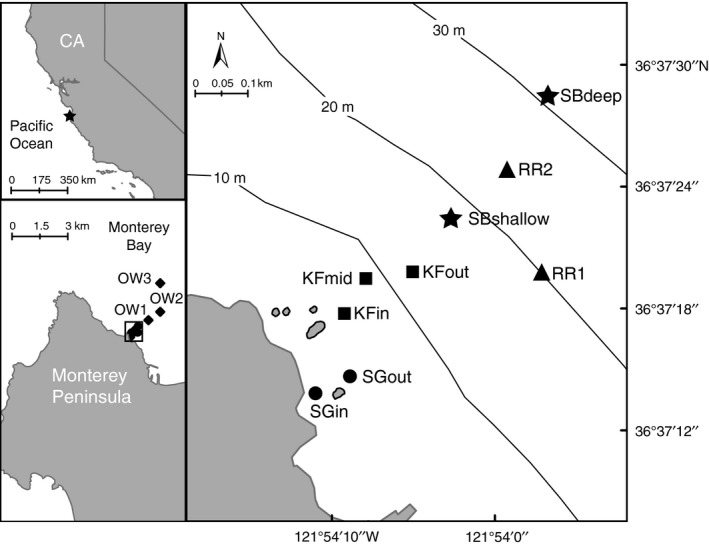
Map of the study region showing the cross‐shore transect located off the Monterey Peninsula in California, USA. Sample sites are grouped by shape: ● = seagrass (SG), ■ = kelp forest (KF), ★ = sandy bottom (SB), ▲ = rocky reef (RR), ♦ = open water (OW). The three open water locations are shown in the left bottom panel. See Table 1 for descriptions of sample sites.

We conducted visual fish SCUBA surveys and collected seawater samples within the Lovers Point‐Julia Platt State Marine Reserve in Monterey Bay adjacent to Hopkins Marine Station of Stanford University on November 13, 2013 (Fig. 1). The 12 survey sites were located within a 2.5 km cross‐shore swath that spanned a depth range of 2–70 m and encompassed a diverse range of habitat types including *Phyllospadix* spp. beds (seagrass), *Macrocystis pyrifera* dominated rocky reef (kelp forest), deeper rocky reef free of *M. pyrifera* (rocky reef) and sandy bottom (Table 1). Within each habitat type, surveys and water sampling were conducted at multiple locations, which in some cases represented distinct microhabitats. Open water sites were outside of safe diving limits and thus only surface water was taken as it could be compared with well‐studied pelagic communities. Surveys and sampling were performed over a 4‐h window from 9:30 to 13:30 (local time). Sites were surveyed using a modified version of the roving diver technique (Schmitt & Sullivan 1996) where experienced divers trained in visual surveys for these habitats (Thompson & Mapstone 1997) ‘roved’ a given site for a predefined period (10 min) while maintaining a constant depth and staying within the prescribed habitat type. Fish were identified at the family level and sized to the nearest 10 cm. Estimated abundance was recorded in one of four log_10_ categories: single (1), few (2–10), many (11–100) and common (101–1000). This technique has been widely used to provide a rapid assessment of the abundance and spatial distribution of fish taxa and has proven robust at providing data on relative abundance and identifying rare species (Schmitt *et al*. 2002), although cryptic and highly mobile species are probably underrepresented. For data analysis, the median count values for each abundance category were used.

**Table 1:** Site‐specific survey details and associated metadata

Site name	Habitat description	Latitude (N)	Longitude (W)	Distance from shore (m)	Depth (m)^a^	Species richness (eDNA)^b^
SGin	Inner seagrass	36°37′13.82″	121°54′11.01″	44	2	16.7 ± 0.6
SGout	Outer seagrass	36°37′14.65″	121°54′8.87″	97	3	11.0 ± 1.0
KFin	Inner kelp forest	36°37′17.72″	121°54′9.29″	140	5	14.0 ± 1.0
KFmid	Mid kelp forest	36°37′19.5″	121°54′7.85″	206	9	13.0 ± 1.7
KFout	Outer kelp forest	36°37′19.85″	121°54′4.932″	262	12	11.3 ± 1.2
SBshallow	Shallow sandy bottom	36°37′22.31″	121°54′2.71″	355	18	8.0 ± 1.0
RR1	Rocky reef	36°37′19.74″	121°53′57.24″	421	21	3.7 ± 0.6
RR2	Rocky reef	36°37′24.84″	121°53′59.29″	470	24	4.7 ± 0.6
SBdeep	Deep sandy bottom	36°37′28.48″	121°53′56.68″	596	30	9.7 ± 1.5
OW1	Open water	36°37′37.77″	121°53′46.54″	978	48	5.3 ± 0.6
OW2	Open water	36°37′49.87″	121°53′35.72″	1436	55	5.7 ± 1.1
OW3	Open water	36°38′29.28″	121°53′19.92″	2665	70	3.0 ± 0.0

a Sampling depth was 1 m from the bottom for all sites except the open water locations where samples were taken 1 m from the surface.

b Mean observed species richness ± SD for the three filter replicates per site.

### Sample collection

At the midpoint of each survey, divers collected a 3‐L water sample at 1 m above the bottom for eDNA using a collapsible plastic bottle (Cubitainer LDPE insert, Hedwin Co., USA). The collection bottles were purchased new for this study and acid washed before use, and each bottle was assigned to a separate sampling site. In addition, water samples were collected at 1 m depth at three sites offshore of the visually surveyed locations (Fig. 1 and Table 1). Divers wore nitrile gloves to reduce possible sample contamination with their own DNA. Collection bottles were closed underwater once full and not opened again until in the laboratory for water filtration. The 3‐L water composite samples were homogenized by inverting and shaking the collection bottles before filtration. Water filtration was performed at Hopkins Marine Station (adjacent to the sampling area) in a laboratory free and with no recent history of any DNA‐based work or fish‐handling. Laboratory benches were sterilized with 10% bleach and the outside of the collection bottles wiped with rnase away (Molecular BioProducts, Inc.) before filtration to reduce the risk of cross‐contamination between samples. Each litre of the 3‐L composite sample was vacuum‐filtered separately using 250 mL disposable analytical test filter funnels (Nalgene, USA) onto 0.22 μm pore size (47 mm diameter) Durapore filters (Millipore, MA, USA) (three filter replicates per site; 36 filter replicates total). Filters were then folded inwards, placed in 2 mL tubes and stored at −80 °C until DNA extraction. Each filter replicate was processed and sequenced separately. A collection blank (1‐L of deionized water brought into the field and bottle uncapped then capped) (*n* = 1) and filtration blanks (1‐L deionized water run through blank filters) (*n* = 3) were included to monitor for contamination.

### Laboratory environment

Sample processing was performed in a laboratory (A. Boehm laboratory, Stanford University) predominantly used for bacterial work. Benchtops were cleaned with 10% bleach and pipettes UV‐irradiated and wiped with rnase away before beginning any molecular work. Filter tips were used for pipetting to further reduce contamination risks. We employed rigorous controls to monitor for contamination at each step of the process, including field, filtration, extraction and PCR blanks. DNA extractions were conducted on a dedicated bench, separated from PCR and post‐PCR work. PCR mastermixes were prepared in a DNA‐free hood and DNA template added on a different laboratory bench as the extractions. All post‐PCR work was performed in a room physically separated from pre‐PCR work.

We have used the same laboratory to process eDNA samples from an aquarium tank in which the majority of amplicons were from sardines (*Sardinops*), tuna (*Thunnus*), menhaden (*Brevoortia*), turkey (*Meleagris*), pig (*Sus*) and human (*Homo*) (Kelly *et al*. 2014). The genera are reported here to highlight the possibility of carry‐over contamination of this study with amplicons from the prior study. We note, however, that amplicons for the two studies were generated approximately 18 months apart, and benches were routinely cleaned with 10% bleach solution during the intervening months. To build the mock communities (see below) we extracted fish tissue samples at a different bench within the same laboratory (these included species that do occur in Monterey Bay, for example rockfish and coho salmon), also plausible sources of contamination.

### DNA extraction

We extracted DNA from each filter using the PowerWater DNA Isolation Kit (MoBio Laboratories, CA, USA). Extraction blanks (*n* = 9) were included for all extractions and run in subsequent PCRs. We also extracted triplicate positive controls consisting of tissue from swordfish, *Xiphias gladius* (DNeasy Blood and Tissue Kit; Qiagen, USA), and amplified these to monitor for the presence of false positives during the PCR and sequencing steps. Two additional positive controls for sequencing included a mix of total DNA extract from 10 species of bony fishes in equimolar concentration (mock community 1), and a mix of total DNA from the same fishes in increasing concentration (mock community 2) (Table S1, Supporting information). Tissue extractions were performed 3 months prior to the filter extractions. DNA extract concentrations were determined using the qubit dsdna hs assay (Invitrogen, CA, USA).

### PCR amplification

We used a published vertebrate‐specific primer set targeting a small region of the mitochondrial DNA 12S rRNA gene (Riaz *et al*. 2011). Primer sequences were F‐5′ ACTGGGATTAGATACCCC and R‐5′ TAGAACAGGCTCCTCTAG, amplifying a ca. 106‐bp gene fragment. We previously validated this primer set in a seawater mesocosm study and found a low false‐negative rate for bony fishes but high false‐negative rate for cartilaginous fishes (Kelly *et al*. 2014). These primers were modified by the addition of specific tags on the 5′ ends to allow for the assignment of sequence reads to the correct sample during bioinformatic processing (Valentini *et al*. 2009). Tags were designed using the oligotag program (Coissac 2012) and consisted of six nucleotides with a Hamming distance of at least three bases between tags. Tags were preceded by nnn (De Barba *et al*. 2014), and the forward and reverse primers for a given sample had identical tags. PCR reactions were carried out using 5 μL DNA extract (1:10 dilutions), 12.5 μL HotStarTaq Plus Master Mix (Qiagen, USA), 1 μL of each primer (10 μm) and 5.5 μL molecular‐biology‐grade water (Sigma‐Aldrich, USA). Eight‐strip PCR tubes with individually attached lids were used instead of 96‐well plates to reduce cross‐contamination between samples. Thermal conditions for PCR were 95 °C for 5 min followed by 40 cycles of 95 °C for 15 s, 55 °C for 30 s and 72 °C for 30 s. Four replicate PCR assays were performed for each filter replicate (*n* = 54) and then pooled. A no template control (NTC) was included for each filter replicate to account for mastermixes with different tagged primer sets. The pooled PCR products were run through a 1.5% agarose gel stained with ethidium bromide to confirm the presence of the 12S target band and clean NTCs and absence of any nonspecific amplification. All NTCs were negative. PCR products were purified and size selected using the Agencourt AMPure XP bead system (Beckman Coulter, USA) and then quantified using the qubit dsdna hs assay (Invitrogen, CA, USA).

### Library prep and DNA sequencing

Tagged PCR products for the 54 samples were pooled in equimolar concentration (20 ng DNA per sample). The concentration of the final pool was 2.33 ng/μL. If a sample did not have at least 20 ng of DNA (e.g. blanks), the entire amount of sample DNA available was added to the pool. 150 ng of DNA from the pool was used for library preparation. Library construction for Illumina sequencing followed the KAPA low‐throughput library prep kit with real‐time library amplification protocol (KAPA Biosystems, MA, USA) in combination with a single nextflex dna barcode (BIOO Scientific, TX, USA) containing the Illumina adapter sequence. A single library was prepared for all tagged PCR products across all samples. Library size and concentration were assessed using a Bioanalyzer with High Sensitivity DNA assay (Agilent Technologies, CA, USA). Sequencing was carried out at the Stanford Functional Genomics Facility on an Illumina MiSeq platform using paired‐end sequencing and a 20% PhiX spike‐in control to improve the quality of low‐diversity samples.

### Sequence analysis

**Figure 2:**
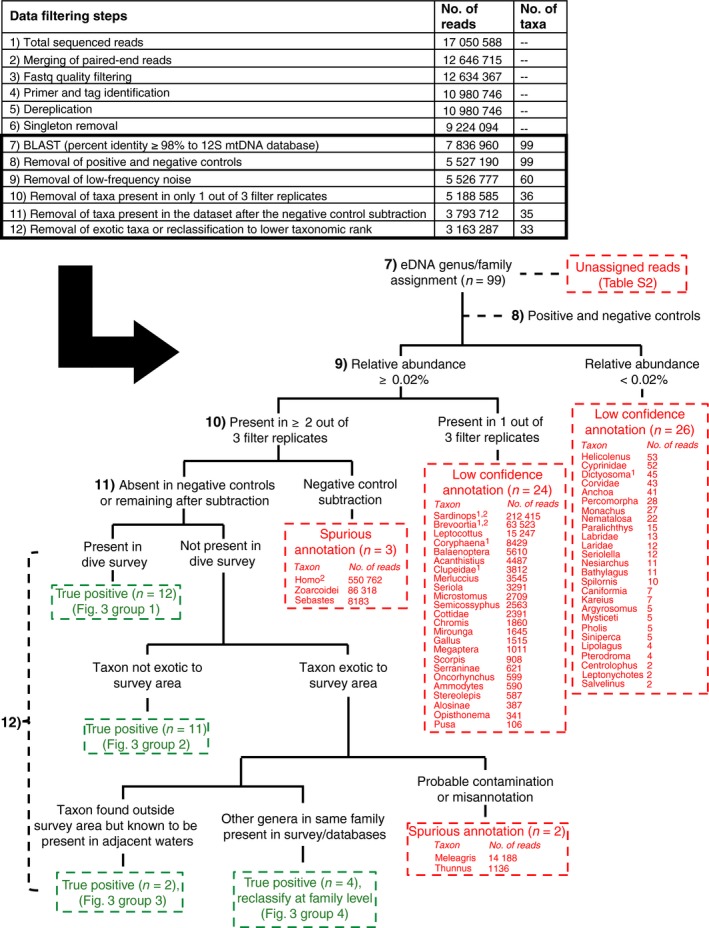
Data analysis framework for processing the eDNA sequence reads. The 12 data‐filtering steps to identify true (green) and false (red) positive assignments are listed along with the number of reads and taxa removed at each step. Steps 7‐12 are presented in a decision tree framework to show the classification scheme of spurious annotations, low‐confidence annotations, and true positives, as well as to list the taxa omitted at each step. ^1^Present in the negative controls (extraction, collection or filtration blanks) but removed prior to step 11. ^2^Probable laboratory contamination but removed prior to step 12.

We employed stringent sequence and taxon filtering parameters in an effort to generate a high‐confidence data set and to repeatably classify true positives and remove false positives resulting from: (i) low‐quality and spurious reads, (ii) low‐confidence annotations and (iii) spurious annotations (Fig. 2). Bioinformatic analyses were implemented with a Unix shell script, which incorporates command line tools as well as calls to third‐party software as follows: Paired‐end reads were first merged with pear v0.9.2 (Zhang *et al*. 2014) using the following parameters: minimum overlap size = 100, maximum assembly length = 161, minimum assembly length = 151, quality score threshold = 15 and *P*‐value = 0.01. Quality filtering was performed using the fastq_filter command in usearch v7.0.1090 (Edgar 2010) with the following parameters: expected number of errors per read = 0.5 and minimum sequence length = 154. Merged reads were demultiplexed by tag sequence using the programming language awk. To minimize the presence of chimeric sequences and tag jumping, only those reads containing the same tag sequence at both the 5′ and 3′ ends were retained (Schnell *et al*. 2015). Reads with homopolymers >7 bases were also omitted. The forward and reverse primers were then removed from the demultiplexed reads using cutadapt v1.4.2 (Martin 2011) allowing for two mismatches in the primer sequence. usearch was used to dereplicate identical reads (−derep_fulllength), remove singletons (−sortbysize), and then cluster into operational taxonomic units (OTUs) at ≥99% identity while further removing chimeras (−cluster_otus). OTUs were compared to a local nucleotide database containing mitochondrial sequences from the National Center for Biotechnology Information (NCBI) using blast+ (Camacho *et al*. 2009). This database—deposited in the Dryad Digital Repository—totalled 12 709 sequences and included the complete mitochondrial genomes as well partial 12S rRNA gene fragments of bony fishes (Actinopterygii), cartilaginous fishes (Chondrichthyes), true seals (Phocidae), sea lions (Otariidae), whales (Cetacea), marine dolphins (Delphinidae), sea otters (*Enhydra*) and birds (Aves) (sequences downloaded September 2014). Default blast parameters were used except for the following modifications: e‐value ≤1e‐20, per cent identity ≥98%, and word size = 24. Taxonomy was assigned to the most specific rank possible (generally family or genus) using the lowest common ancestor (LCA) algorithm in megan v5.5.3 (default settings except for: min score = 150, top per cent = 2) (Huson & Weber 2013). Reads with no matches to sequences in our 12S database were not included in subsequent analyses but were annotated using blast against the full NCBI nucleotide database returning up to 500 hits per query sequence at an e‐value threshold of ≤1e‐20 to determine their probably identities. From these results, we assigned taxonomy from the hits with the lowest e‐value at levels of ≤1e‐40, ≤1e‐35, ≤1e‐30, ≤1e‐25 and ≤1e‐20 (Table S2, Supporting information).

We identified two types of problematic annotations, which we considered to be ‘false’ positives: low‐confidence annotations and spurious annotations. Low‐confidence annotations are defined here as high‐quality annotated reads passing the previous filtering steps that have high‐confidence blast matches but may be inaccurate due to being (i) present at frequencies below the low‐frequency noise threshold set by the positive controls or (ii) present in only one of the three filter replicates per sample. We sequenced the positive controls included in this experiment to establish filtering parameters for low‐frequency noise (De Barba *et al*. 2014). We set a discard abundance, or low‐frequency noise, threshold at 0.02%. This threshold represents the abundance of the spurious taxon (i.e. a taxon other than our control species) with the highest abundance in the positive controls, and for each sample, we discarded all taxa whose frequency was below 0.02% (Fig. 2). The criterion to exclude taxa if present in only one of the three filter replicates balanced the need to identify PCR/sequencing errors and contamination against the desire to detect low‐concentration DNA; our analysis assumes that a taxon occurring in only one of three filter replicates is more likely the former than a true (but rare and therefore stochastic) signal. While excluded from the remaining analysis, the identities of low‐confidence annotations (taxa and read counts) are shown in Fig. 2.

Spurious annotations are also high‐quality reads passing the previous filtering steps that have high‐confidence blast matches but are probably inaccurate due to being (i) present in the negative controls or (ii) exotic to the survey area and adjacent waters. We additionally sequenced the negative controls (i.e. field and extraction blanks) included in this experiment to identify sequences that were likely the result of contamination. The number of sequences of each taxon present in the respective negative control was subtracted from the sequence abundance of that taxon in the field sample (Nguyen *et al*. 2015). All remaining annotated reads were either classified as true positives or spurious annotation due to being exotic to the survey area. Taxa classified as true positives were either also seen in the visual surveys or are likely present in the survey area based on other biological information including the kelpforest Database (Beas‐Luna *et al*. 2014) and survey data from historical monitoring at Hopkins Marine Station and California waters (Miller & Lea 1972). The kelpforest Database is a repository containing the identities of species present in nearshore kelp forest ecosystems of the eastern Pacific Ocean, with a focus on central and southern California. Taxa not known to be present in the survey area but are present in adjacent waters or have other genera in the same family that are known to be present in the survey area or database were also classified (at the family level for the latter case) as true positives. Remaining taxa were classified as exotic to the survey area and excluded from the analysis. Thus the use of extrinsic natural‐history data provided an independent means of assessing eDNA error rates for the study.

### Statistical analyses

eDNA sequence counts were normalized with the r package deseq2 v1.6.2 (Love *et al*. 2014). This method accounts for differential sample depth (correcting for uneven numbers of reads per tag) and is appropriate for normalizing high‐variance data sets from high‐throughput sequencing (McMurdie & Holmes 2014). In addition, we created a presence/absence version of the data set to compare to the count data.

We assessed pairwise differences in annotated sequence abundances between all pairs of sample sites and habitat types using a Kruskal–Wallis test with Bonferonni correction. The Kruskal–Wallis test was also used to test for differences in whole‐community membership, for example differences in annotated read abundances calculated over all habitats simultaneously, between and within habitat types and between filter replicates. We further analysed beta‐diversity and community composition in both versions of the data set (normalized counts and presence/absence) with the betadiver, adonis, hclust and metaMDS functions using the vegan package v2.2.0 of r (Oksanen *et al*. 2008). We used adonis to do a nonparametric analysis of variance (permanova) of Bray‐Curtis dissimilarities among samples, testing for significance using 200 permutations. Nonmetric multidimensional scaling (NMDS) was performed using the Bray‐Curtis dissimilarity index with metaMDS.

## Results

### Sequence data processing

Sequencing of vertebrate 12S mitochondrial eDNA for all samples generated over 17 million paired‐end reads with a relatively uniform read distribution across primer tags (195 091 ± 24 448 reads per tag for field samples) (Fig. S1, Supporting information). We employed stringent sequence filtering parameters as described above, generating a high‐confidence data set consisting of 3.16 million, 106‐bp reads (Fig. 2). Sequences excluded from the analysis due to not having any taxonomic matches at the specified blast or megan thresholds (*n* = 860 907) were subsequently annotated against the full NCBI nucleotide database at varying e‐value thresholds and consisted mainly of reads annotated as *Epinephelus* (*n* = 431 987) and *Rhacochilus* (*n* = 108 437) (Table S2, Supporting information). Only two spurious taxa (*Gallus* and *Homo*) were present in the positive controls (i.e. mock communities and swordfish tissue), and the higher relative abundance for *Gallus* (0.02%) was used to set the low‐frequency noise threshold across the entire data set (Table S1, Supporting information). The source of these two spurious taxa in the mock communities may be contamination from DNA extraction and/or PCR reagents during production in the manufacturer's laboratories, or laboratory processing (Champlot *et al*. 2010). The low‐frequency noise filter removed 26 of 99 taxa identified by blast (Fig. 2). An additional 24 taxa were removed due to their presence in only one of the three replicates per sample (Fig. 2). After the previous filtering steps, three taxa remained that were also present in the negative controls (*Homo*,* Sebastes* and Zoarcoidei, a perciform suborder containing gunnels and similar fishes). Subtraction of the number of sequence reads for each of these genera in the negative controls from their sequence abundances in each field sample resulted in the exclusion of *Homo* (27.9% of total annotated reads) from the data set. The final filtering steps removed two more taxa that were classified as exotic to the survey area and probable contamination or misannotation (*Meleagris* and *Thunnus*). Four genera not known to be in the area (*Epinephelus*,* Etropus, Odontesthes* and *Plectobranchus*) were reassigned to the family level due to other family members being present in the survey area and in the kelpforest Database; these cases were probably misannotations due to incomplete coverage of these groups in GenBank. The final filtered data set contained 33 unique taxa, with 26, six and one taxa annotated at the genus, family and suborder levels, respectively.

### eDNA vs. visual surveys

**Figure 3:**
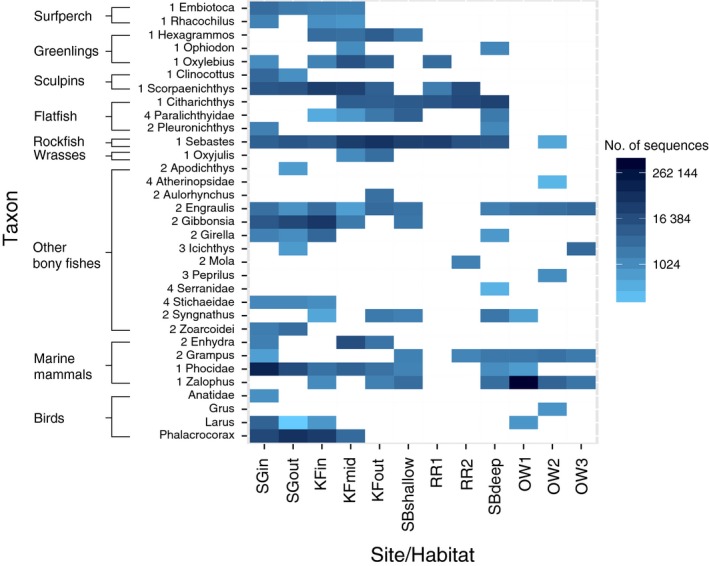
Heatmap showing the mean normalized counts of taxa detected with eDNA at each sample site. Means represent the average of the three filter replicates per sample site. See Table 1 for descriptions of sample sites. Numbers in front of taxon names correspond to the classification groups for true positives as defined in Fig. 2. SG, seagrass; KF, kelp forest; SB, sandy bottom; RR, rocky reef; OW, open water.

Visual surveys revealed seven fish groups and two pinnipeds (12 taxa) across all habitats, except the open water habitat where visual data were not collected (Table S3, Supporting information). Of the 12 visually observed taxa, 11 were also detected with eDNA (Fig. S2, Supporting information; false‐negative rate = 8.3% relative to visual survey). Gobies were the lone taxon seen visually but not detected with eDNA. eDNA detected 18 additional taxa classified as true positives based on their known presence in the survey area (Fig. 3), more than doubling the total number of vertebrates surveyed.

**Figure 4:**
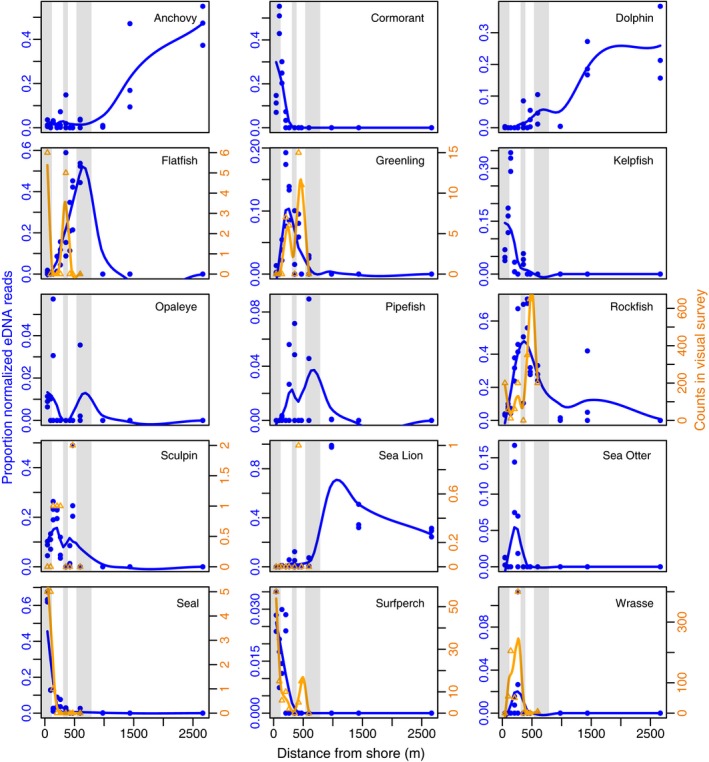
Spatial trends in eDNA and visual count data across the transect, habitats and microhabitats. Panels are shown for those taxa that were present in the visual surveys in addition to those taxa whose summed proportional abundance across all samples was >10%. eDNA counts for the three filter replicates are plotted for each sample site. eDNA counts were significantly associated with habitat for all taxa listed (KW,* P* < 0.05) (see Table S4, Supporting information). Visual counts are included for taxa seen on the dive surveys (no visual surveys for the open water sites). Loess curves for visual counts are only included for taxa with >15 counts total across all surveyed sites and are not best‐fit lines. Plot background (grey and white shading) distinguishes the following habitat types, moving away from shore: seagrass, kelp forest, shallow sandy bottom, rocky reef, deep sandy bottom and open water. See Fig. S3 (Supporting information) for distance from shore plotted on a log(x) scale. (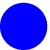) proportion normalized eDNA reads; (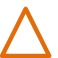) visual counts.

The spatial trends of visual and eDNA counts were highly concordant for the major fish groups surveyed (Fig. 4 and Fig. S3, Supporting information). We were unable to detect a difference in the spatial distribution of eDNA vs. visual survey counts for any of the taxa occurring in both data sets (Kolmogorov‐Smirnov tests, *P *>* *0.05), although our power to detect differences was limited by small sample sizes in the visual data. Peak eDNA abundance and survey counts co‐occurred within ca. 100 m for species as diverse as rockfish, wrasses, surfperch and seals. Flatfish—which can be difficult to survey visually—had contrasting visual and eDNA counts, with eDNA peaking in the sandy bottom habitat.

To evaluate whether PCR amplification bias among taxa due to primer mismatch may have impacted detection and sequence abundance, we aligned the 12S primer sequences with the primer‐binding sites for species known to be present in kelp forest ecosystems of Monterey Bay as well as species in the mock communities (Fig. S4, Supporting information). All of the taxa that we detected (i.e. bony fishes and marine mammals) appear to have fewer than two mismatches and if present are located at the 5′ of the reverse primer‐binding site which is less likely to result in decreased primer‐binding efficiency relative to the 3′ end (Bru *et al*. 2008; Stadhouders *et al*. 2010). Failure to detect some additional taxa, such as cartilaginous fishes, may be due to known mutations in the primer sites for those taxa (Fig. S4, Supporting information). Despite widespread variation in sequence counts across species in mock community 1 (equimolar) and a weak relationship between DNA concentration and sequence count in mock community 2 (increasing concentration) (Table S1, Supporting information), the mock communities did not show strong evidence for decreased amplification efficiency due to primer bias.

### Spatial distribution of eDNA abundance

Spatial trends in taxon‐specific eDNA were strongly consistent with *a priori* expectations given known species distributions (Fig. 4 and Fig. S3, Supporting information). Taxa were nonrandomly associated with habitat and distance from shore [Kruskal–Wallis (KW) test, *P *<* *0.05], and many exhibited clear peaks in expected habitats (eight taxa significant at *P* < 0.05; three taxa at *P* < 0.01; 15 taxa at *P* < 0.001) (Table S4, Supporting information, All Habitats). For example, the eDNA of rockfish and other kelp forest species such as greenlings (*Hexagrammos*,* Ophiodon*,* Oxylebius*), wrasses (*Oxyjulis*) and sea otters (*Enhydra*) peaked in those species’ core habitats—kelp forest and rocky reef—and decreased in the seagrass and open water samples. Similarly, nearshore taxa including surfperch (*Embiotoca* and *Rhacochilus*) and cormorants (*Phalacrocorax*) were most abundant in the seagrass and absent in habitats offshore. Pinnipeds peaked in seagrass (Phocidae, seals) and in an open water sample (*Zalophus*, sea lions). Anchovies (*Engraulis*) and dolphins (*Grampus*) were nearly absent inshore but peaked offshore. There was more sampling variation in taxon abundances between habitat types than within habitat types (Table S4, Supporting information).

### Patterns of community composition

**Figure 5:**
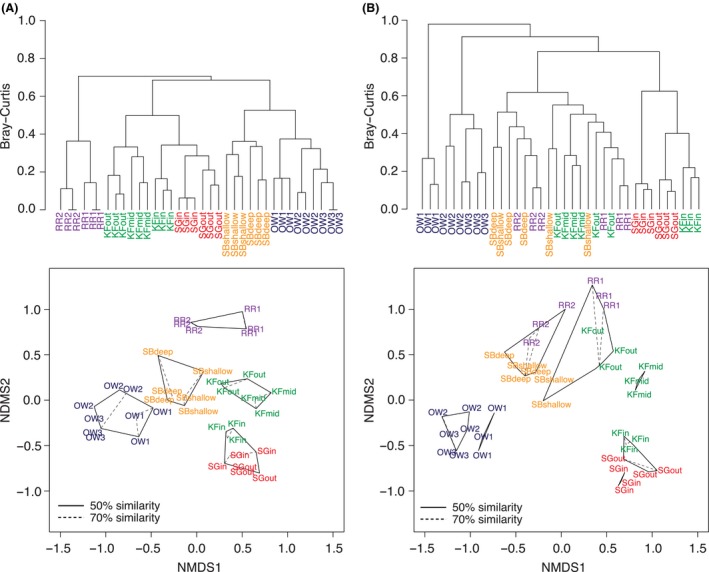
Hierarchical clustering dendrograms and NMDS ordination of the similarity in vertebrate eDNA community composition across habitats and microhabitats. Dendrograms and NMDS plots are shown for (A) binary presence/absence data and (B) normalized counts generated using the deseq2 package. Clustering is based on group‐average linkage from Bray‐Curtis similarity. Clusters at similarity levels of 50% (outer solid polygon) and 70% (inner dashed polygon) are superimposed on the NMDS plots. Labels refer to filter replicates (see Table 1 for descriptions) and are coloured by habitat type. SG, seagrass; KF, kelp forest; SB, sandy bottom; RR, rocky reef; OW, open water.

Between‐site differences accounted for 92.2% of variance in the data set [permutational multivariate analysis of variance (permanova) using distance matrices *P* < 0.005]. Between‐habitat, within‐habitat and filter replicate eDNA variance was 76.3%, 15.9% and 7.8%, respectively (permanova,* P* < 0.005). Vertebrate communities associated with microhabitats of seagrass, kelp forest, rocky reef and open water habitats were distinguishable using eDNA (Table S5A, Supporting information); thus this method was able to distinguish among communities separated by as little as ~60 m, the smallest spatial interval sampled. Clustering and ordination of eDNA community composition based on presence/absence data distinguished each habitat type from one another (Fig. 5A), but count data showed overlap between sandy bottom, rocky reef and outer kelp forest communities (Fig. 5B). Of the 15 pairwise habitat comparisons, 14 were significantly different (KW, *P* < 0.05, *R* ^2^ = 0.473–0.771) (Table S5B, Supporting information). Filter replicates from the same location showed low variability in observed species richness (Table 1). Overall, the different habitats displayed unique taxonomic assemblages, and vertebrate species richness decreased with distance from shore (Table 1).

## Discussion

Our survey of marine vertebrate diversity sheds light on the spatial resolution and error rates of field‐based eDNA studies by comparing to conventional visual surveys and sampling across multiple nearshore habitats. eDNA in seawater samples offered accurate, relatively fine‐scale spatial resolution (60–100 m) of vertebrate communities across multiple habitats in a highly dynamic nearshore environment, and compared favourably to traditional visual surveys, often the standard of ecological monitoring.

We found spatially concordant trends in individual species’ eDNA and visual surveys, and taxa detected in each habitat with eDNA reflected highly local community composition. The eDNA is therefore likely to be primarily of local origin and the probability of detecting nonresident species decreases with distance from those species distributions, consistent with the only other field eDNA survey of marine fishes to date (Thomsen *et al*. 2012a). We found significant differences in eDNA community composition and relative abundance of taxa between adjacent habitat types and by distance from shore within a habitat type, indicating that sampling locations in close proximity (~60 m) had distinct eDNA assemblages. If physical processes (e.g. advection, diffusion) drive the dispersal patterns of eDNA, we would expect the eDNA data to be decoupled from the visual surveys and independent of habitat type; conversely, if biological processes (e.g. generation and degradation of eDNA) are more influential, we would expect species’ spatial patterns to differ systematically. Our results support the latter hypothesis, although the detection probability in this study system may be high due to local oceanography at the time of sampling, the retentive properties of kelp forests (Gaylord *et al*. 2012), or both. We note, too, that differences in community composition and taxon abundance between the open water vs. nearshore habitats may also be due to depth as open water samples were collected at the surface while all other habitat samples were collected 1 m from the bottom. The greatest depth difference between these samples was 29 m.

This study also illustrates the uncertainties surrounding assessing error rates of detection in an open ecosystem. Factors including the number of sample replicates and gene markers, sequence misannotation, contamination, data‐filtering strategy and ecology and hydrodynamics impacted our estimates of error, and we note that most habitats do not have the advantage of being as extensively documented as Monterey Bay. For eDNA to be practical for biological monitoring, the method must have low error rates of detection in the field. We quantified error rates at several key steps (Fig. 2): First, we conducted visual fish surveys in conjunction with eDNA sampling to test for concordance between taxonomic identity, geographic location and sequence data. Second, we sequenced 1‐L replicates for each composite 3‐L sample to assess sample variability among replicate field samples. Third, we included internal controls to enable removal of low‐frequency noise and any contaminant reads from the data set (De Barba *et al*. 2014). Fourth, we employed stringent sequence filtering parameters to identify false‐positive assignments and remove spurious reads resulting from PCR errors, sequencing errors or carry‐over or cross‐contamination, generating a conservative, high‐confidence data set. Lastly, we filtered for geographic relevance, removing any remaining taxa passing the previous filters that were exotic to the survey area or vicinity or not present in the kelpforest Database. We obtained a low false‐negative rate of eDNA detection relative to the visual surveys (1/12 taxa). eDNA detected more than twice the number found in the visual survey (29 eDNA, 12 visual) due to the inclusion of cryptic species (e.g. flatfish, gunnel, kelpfish) not easily seen with visual methods and/or advection of DNA from species outside the immediate survey area (e.g. medusafish, Pacific Butterfish). While our study design did not allow for occupancy modelling due to one sampling event per location and insufficient replication to detect rarer taxa (Schmidt *et al*. 2013; Ficetola *et al*. 2015), this approach may improve low detection rates and the reliability of eDNA metabarcoding studies.

It was not possible—and neither was it our objective—to estimate the number of false‐negative or false‐positive detections relative to the ‘true’ species composition of the survey area. Given the true species composition is unknown, we acknowledge that our conservative sequence filtering may have decreased the number of species detected. For example, taxa known to be present in and around the survey area including *Leptocottus* (Pacific staghorn sculpin), *Semicossyphus* (sheephead wrasse), *Merluccius* (hake) as well as the whale genera *Megaptera* (humpback whale) and *Balaenoptera* (blue, fin or minke whale), were detected with eDNA but classified as false negatives due to low abundance or presence in only one of three filter replicates. A similar majority‐rules approach for replicates has been implemented in other metabarcoding studies (Giguet‐Covex *et al*. 2014; Ficetola *et al*. 2015). When eDNA is rare variability across replicates may be expected, but our approach aimed to balance capturing sample heterogeneity with minimizing spurious assignments due to PCR or sequencing errors, primer tag bias (J. L. O'Donnell, R. P. Kelly, N. C. Lowell & J. A. Port, unpublished data) and/or contamination. Sample heterogeneity in this study underscores the need for a larger number of replicates (perhaps as much as >8), especially for species with low detection probabilities and field studies lacking ground‐truthing data (Ficetola *et al*. 2015), but see Lahoz‐Monfort *et al*. 2015. However, we emphasize that the goal of this study was to survey and compare whole communities, not to detect rare taxa. For such applications, more sensitive, targeted protocols such as quantitative real‐time PCR (qPCR) may be more appropriate. Additionally, multiple gene markers may improve detection rates in metabarcoding studies (Evans *et al*. 2015) due to increased likelihood of finding matches in sequence databases and reduced primer bias. For example, the absence of cartilaginous fishes in our data set may likely be due to two mismatches present at the 3′ end region of the forward primer‐binding site that may inhibit amplification (Fig. S4, Supporting information; Kelly *et al*. 2014).

Taxa that were present in the eDNA data but absent in the dive surveys and local taxonomic databases may result from: (i) sequence misannotation due to sequencing errors or incompleteness of the NCBI nucleotide database, (ii) DNA derived from field or laboratory contamination, (iii) movement of eDNA sourced from species located outside the survey area, and/or (iv) resuspension of sedimentary eDNA (Turner *et al*. 2015). The latter two sources were not directly investigated in this study. Because of the shorter length of the 12S locus (~106 bp), sequencing errors are more likely to lead to misannotation for closely‐related taxa. This was evident for taxa removed due to low‐confidence annotation. For example, true seals (Phocidae) were common in our data set as expected but genera of this family with lower sequence counts (*Pusa*,* Monachus* and *Leptonychotes*) are polar or subtropical in distribution. Similarly, many of the other low‐confidence annotations were uncommon or not known to be in the survey area but had other family members present in the system or kelpforest Database.

Regarding contamination, *Sardinops* and *Brevoortia* (family Clupeidae) were both present in the negative controls and had high sequence counts in the combined data set (212 415 and 63 523, respectively). These genera are potential carry‐over contaminants from previous experiments. Our data‐filtering steps removed these two taxa before even filtering specifically for probable carry‐over laboratory contamination though (Fig. 2). To minimize the level of potential human contamination in our samples, we originally processed the samples in this study using a human blocking primer designed to bind to the target 12S gene region and reduce amplification of human DNA. While human DNA was effectively blocked, there was also unexpected and unpredictable blocking of fishes we expected to find (e.g. rockfish and halibut) based on PCR amplification and sequence data (data not shown). We therefore subsequently amplified and sequenced the samples without a human blocker. Similar amplification biases have been seen in other studies when using blocking primers with universal primers (Pinol *et al*. 2015). *Meleagris* and *Thunnus* passed all data‐filtering steps including absence in negative controls, but were not relevant to the geographic scope of the study area and so were classified as false positives stemming from possible contamination or misannotation. *Meleagris*, like *Gallus* and *Homo*, may be due to contamination in DNA extraction or PCR reagents during production in the manufacturer's laboratories. While *Thunnus* is not present in the survey area, other scombrids are known to be present which could lead to misannotation due to high sequence similarity across species in the 12S gene region. It is possible that *Thunnus* and other taxa excluded as false positives may also be sourced from the Tuna Research and Conservation Center or Monterey Bay Aquarium which deposit effluent in proximity to the survey area.

Biological and methodological biases are hurdles to estimating species abundances from eDNA (Pompanon *et al*. 2012), as evidenced by a weak relationship between DNA concentration and sequence abundance in our mock communities (Table S1, Supporting information). For this reason, we compared sequence abundances between sampling locations only within individual taxa, rather than comparing across taxa. Our analysis assumes that eDNA fragments from a given taxon experience the same extraction, amplification and sequencing biases—and same mtDNA copy number per cell—regardless of the location from which they were collected. One exception was the community‐level analysis, which unavoidably required cross‐taxon comparisons; however, presence/absence eDNA data differentiated vertebrate communities even more definitively (Fig. 5A). Tissue‐ or species‐correction factors (Thomas *et al*. 2014), designing and optimizing generic primers (Thomsen & Willerslev 2015), and PCR‐free approaches (Zhou *et al*. 2013) are under investigation and may help to link eDNA abundance to organism abundance in the future. Understanding variability in DNA shedding rates across species will also strengthen quantitative estimates (Maruyama *et al*. 2014; Klymus *et al*. 2015).

We have shown that even in a nearshore environment subject to wave action and mixing, eDNA may substantially improve upon traditional survey methods and ecologists’ power to monitor the dynamics of whole animal communities. Of particular management relevance, the technique reveals spatial trends in the presence and abundance of iconic species in kelp forest ecosystems important for fisheries and ecosystem health (e.g. rockfish, lingcod, sea otters, cabezon and striped surfperch). Temporally, this study is only a snapshot; further studies will address the stability of eDNA measurements over time in dynamic systems. Seasonal variation in currents, temperature and species abundance patterns as well as variability in fish fauna diversity over the diurnal cycle and water column are important factors influencing both eDNA and visual detection. In addition, ecological and hydrological factors influencing eDNA detection rates such as eDNA shedding rates, fate and transport, and degradation rates require more attention (Jerde & Mahon 2015). Several studies have already shown eDNA to be more cost‐effective than traditional monitoring methods (Thomsen & Willerslev 2015). With further validation and refinement, eDNA holds promise as a more comprehensive approach to large‐scale environmental monitoring.

J.A.P., R.P.K., K.J.N., S.Y.L. and P.R.L. designed the study. K.J.N., S.Y.L. and P.R.L. performed field research. J.A.P. and O.C.R. conducted the laboratory work. J.A.P., J.L.O. and R.P.K. analysed the data. J.A.P., R.P.K. and K.M.Y. wrote the manuscript.

## Data accessibility

Nucleotide sequences: NCBI SRA: SRP065606. Mitochondrial nucleotide blast database (in fasta format) and raw taxon table: Dryad doi:10.5061/dryad.nf578. Results of the visual surveys are archived as Supporting Information for online publication. Unix pipeline used for processing sequence data: https://github.com/jimmyodonnell/banzai/.

## Supporting information


**Fig. S1.** Number of reads per tag assigned to field samples, positive controls (i.e. mock communities and swordfish tissue) and negative controls (i.e. filtration and extraction blanks) after demultiplexing.
**Fig. S2.** Venn diagram of bony fish taxa and marine mammals as detected by eDNA vs. visual survey for the combined sample sites.
**Fig. S3.** Spatial trends in eDNA and visual count data across the transect and habitats shown on a log(x) scale.
**Fig. S4.** Sequence alignment of the 12S rRNA primer‐binding sites for taxa present in Monterey Bay and the mock community.

**Table S1.** (A) Composition of the artificial communities used as positive controls. The two communities contained either equal (community 1) or increasing (community 2) concentrations of tissue DNA. (B) Sequence counts for each taxon present in the three positive controls.
**Table S2.** Taxonomic annotation and read counts for sequences not matching species in our 12S database at specified blast and megan thresholds.
**Table S3.** Visual fish survey counts for each sample site.
**Table S4.** Pairwise‐comparisons of taxon abundance between (A) habitat types and (B) sample sites within the same habitat.
**Table S5.** Statistical tests of beta‐diversity (A) between sites within the same habitat and (B) between habitats (*R* ^2^ values).
