# Supplementary material for: Assessing vertebrate biodiversity in a kelp forest ecosystem using environmental DNA
Source: Mol Ecol. 2015 Dec 24;25(2):527–41. doi: 10.1111/mec.13481 (PMC4737306; doi:10.1111/mec.13481)
Supplement: Supplementary file 1 — Fig. S1. Number of reads per tag assigned to field samples, positive controls (i.e. mock communities and swordfish tissue) and negative controls (i.e. filtration and extraction blanks) after demultiplexing. Fig. S2. Venn diagram of bony fish taxa and marine mammals as detected by eDNA vs. visual survey for the combined sample sites. Fig. S3. Spatial trends in eDNA and visual count data across the transect and habitats shown on a log(x) scale. Fig. S4. Sequence alignment of the 12S rRNA primer‐binding sites for taxa present in Monterey Bay and the mock community. [file MEC-25-527-s001.docx]

**Fig. S1.** Number of reads per tag assigned to field samples, positive controls (i.e. mock communities and swordfish tissue) and negative controls (i.e. filtration and extraction blanks) after demultiplexing. The sample size listed in parentheses corresponds to the number of tags for the category (e.g. 3 filters per field site x 12 field sites =36 tags; positive controls also tagged in triplicate). Boxes encompass the interquartile range (IQR), lines extend to 1.5IQR, and dots represent outliers. The outlier in the negative controls (>400,000 reads) corresponds to a unique sequence (+/- 1 bp variants) matching human.

**Fig. S2.** Venn diagram of bony fish taxa and marine mammals as detected by eDNA versus visual survey for the combined sample sites. Numbers indicate taxon counts.

**
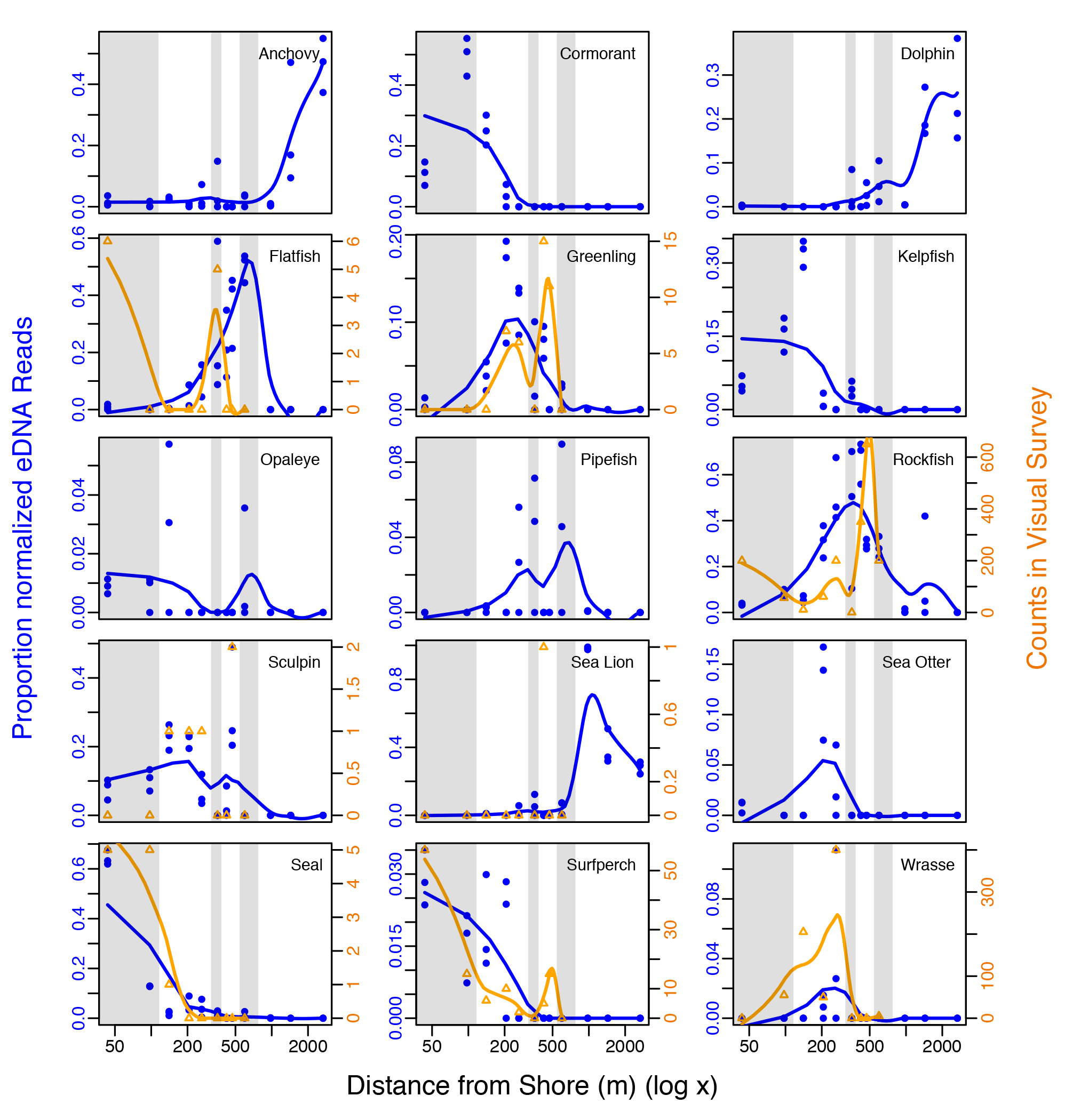
**

**Fig. S3.** Spatial trends in eDNA and visual count data across the transect, habitats, and microhabitats shown on a log(x) scale. The log(x) scale emphasizes differentiation of counts in the non-open water habitats. Panels are shown for those taxa that were present in the visual surveys in addition to those taxa whose summed proportional abundance across all samples was >10%. eDNA counts for the three filter replicates are plotted for each sample site. eDNA counts were significantly associated with habitat for all taxa listed (KW, P<0.05) (see Table S4, Supporting Information). Visual counts are included for taxa seen on the dive surveys (no visual surveys for the open water sites). Loess curves for visual counts are only included for taxa with >15 counts total across all surveyed sites and are not best-fit lines. Plot background (grey and white shading) distinguishes the following habitat types, moving away from shore: seagrass, kelp forest, shallow sandy bottom, rocky reef, deep sandy bottom, and open water. () proportion normalized eDNA reads; () visual counts.

**Fig. S4.** Sequence alignment of the 12S rRNA primer-binding sites for taxa present in Monterey Bay and the mock community. Nucleotide mismatches are highlighted in black. NCBI accession numbers follow each taxon name.
